# Supplementary material for: Substitution of surgical care within benign gynaecology during COVID-19: waste of a good crisis? – a quantitative longitudinal study in the Netherlands
Source: BMJ Open. 2025 Aug 4;15(8):e100357. doi: 10.1136/bmjopen-2025-100357 (PMC12359422; doi:10.1136/bmjopen-2025-100357)
Supplement: Author's manuscript [file bmjopen-2025-100357.draft_revisions.pdf]

BMJ Open is committed to open peer review. As part of this commitment we make the peer review history of every article we publish publicly available.

When an article is published we post the peer reviewers' comments and the authors' responses online. We also post the versions of the paper that were used during peer review. These are the versions that the peer review comments apply to.

The versions of the paper that follow are the versions that were submitted during the peer review process. They are not the versions of record or the final published versions. They should not be cited or distributed as the published version of this manuscript.

BMJ Open is an open access journal and the full, final, typeset and author-corrected version of record of the manuscript is available on our site with no access controls, subscription charges or pay-per-view fees (http://bmjopen.bmj.com).

If you have any questions on BMJ Open's open peer review process please email info.bmjopen@bmj.com

BMJ Open

Substitution of Surgical Care within Benign Gynaecology during Covid-19: Waste of a Good Crisis? – A quantitative longitudinal study in the Netherlands

Journal:	*BMJ Open*
Manuscript ID	bmjopen-2025-100357
Article Type:	Original research
Date Submitted by the Author:	07-Feb-2025

Complete List of Authors:	Velthuijs, Eva; Amsterdam UMC Locatie AMC, Gynaecology & Obstetrics<br>Ismail, Ismail; Vrije Universiteit Amsterdam, Health economics<br>Koolman, Xander ; VU Amsterdam<br>de Leeuw, Robert ; Amsterdam UMC Locatie AMC, Department of Gynaecology and Obstetrics<br>Hehenkamp, Wouter; Amsterdam UMC Locatie AMC, Obstetrics and Gynaecology

Keywords:	COVID-19, Delivery of Health Care, Integrated, GYNAECOLOGY

SCHOLARONE™
Manuscripts

I, the Submitting Author has the right to grant and does grant on behalf of all authors of the Work (as defined in the below author licence), an exclusive licence and/or a non-exclusive licence for contributions from authors who are: i) UK Crown employees; ii) where BMJ has agreed a CC-BY licence shall apply, and/or iii) in accordance with the terms applicable for US Federal Government officers or employees acting as part of their official duties; on a worldwide, perpetual, irrevocable, royalty-free basis to BMJ Publishing Group Ltd ("BMJ") its licensees and where the relevant Journal is co-owned by BMJ to the co-owners of the Journal, to publish the Work in this journal and any other BMJ products and to exploit all rights, as set out in our licence.

The Submitting Author accepts and understands that any supply made under these terms is made by BMJ to the Submitting Author unless you are acting as an employee on behalf of your employer or a postgraduate student of an affiliated institution which is paying any applicable article publishing charge ("APC") for Open Access articles. Where the Submitting Author wishes to make the Work available on an Open Access basis (and intends to pay the relevant APC), the terms of reuse of such Open Access shall be governed by a Creative Commons licence – details of these licences and which Creative Commons licence will apply to this Work are set out in our licence referred to above.

Other than as permitted in any relevant BMJ Author's Self Archiving Policies, I confirm this Work has not been accepted for publication elsewhere, is not being considered for publication elsewhere and does not duplicate material already published. I confirm all authors consent to publication of this Work and authorise the granting of this licence.

For peer review only

Title

Substitution of Surgical Care within Benign Gynaecology during Covid-19: Waste of a Good Crisis? – A quantitative longitudinal study in the Netherlands

Authors

E.L.M. Velthuijs (MD)[1], I. Ismail (Msc)[2], A.H.E. Koolman (PhD)[2], R.A. de Leeuw (MD, PhD)[1], W.J.K. Hehenkamp (MD, PhD)[1]

1. Department of Obstetrics and Gynaecology, Amsterdam UMC, Location VU/AMC, Amsterdam, The Netherlands

2. Department of Health Economics, Vrije Universiteit, Amsterdam, The Netherlands

Corresponding author:

E.L.M. Velthuijs

Department of Obstetrics and Gynaecology, Amsterdam UMC, Meibergdreef 9, Amsterdam 1105 AZ, The Netherlands

Email: e.velthuijs@amsterdamumc.nl

Manuscript word count: 3492

For peer review only - http://bmjopen.bmj.com/site/about/guidelines.xhtml

KEY WORDS

COVID-19, delivery of healthcare, gynecologic surgical procedure

For peer review only

For peer review only - http://bmjopen.bmj.com/site/about/guidelines.xhtml

ABSTRACT

Importance: During the COVID-19 pandemic, the capacity for elective surgical care was severely restricted, creating opportunities for long-sought substitution of care and prioritization of high-value care.

Objective: To examine the impact of the COVID-19 pandemic on the substitution of surgical procedures in benign gynaecology in the Netherlands.

Design: Quantitative longitudinal study evaluating the effects of the COVID-19 pandemic.

Setting: Nationwide healthcare delivery was analysed across six benign gynaecological pathways from 2016 to 2022 using Vektis and Dutch Hospital Data (DHD), accessed via Statistics Netherlands (CBS).

Participants: The study focused on six benign gynaecological pathways classified using Dutch Diagnosis Treatment Combinations (DTCs): heavy menstrual blood loss (G11), uterine fibroids (G15), endometriosis (G17), prolapse (G25), infertility treatment (F11), and first trimester pregnancy complications (Z12). Patients receiving care within these pathways between January 1, 2016, and December 31, 2022, were included. Exclusions applied to patients under 18 years old and, within the menstrual disorder pathway, patients over 51 years old to exclude most postmenopausal blood loss cases where no alternative treatment applies.

Intervention or Exposure: Cohorts from the initial pandemic year (2020) were compared to four pre-pandemic cohorts (2016–2019) and late-pandemic (2021) and post-pandemic (2022) cohorts.

Main Outcomes and Measures: The primary outcome was the trend in the total number of patients in surgical and non-surgical procedure groups across cohort periods. Secondary outcomes included trends within individual pathways.

For peer review only - http://bmjopen.bmj.com/site/about/guidelines.xhtml

Results: The analysis identified a significant reduction in benign gynaecological care during 2020, with an 18.3% decrease in surgical procedures and a 6% decrease in non-surgical procedures across all pathways. Non-surgical procedures resumed pre-pandemic trends by mid-2020, whereas surgical procedures did not return to baseline levels.

Conclusions and Relevance: The COVID-19 pandemic significantly disrupted both surgical and non-surgical procedures within benign gynaecological pathways. Reduced care uptake during the pandemic waves was not recovered but instead forgone. The reduction in surgical procedures did not correspond with increased use of non-surgical alternatives. Future research should prioritize evaluating the long-term impacts of this disruption on patients and society.

Trial Registration: Not applicable.

For peer review only

For peer review only - http://bmjopen.bmj.com/site/about/guidelines.xhtml

STRENGTHS AND LIMITATIONS OF THIS STUDY

- The large dataset spanning allows a detailed longitudinal insight into gynaecological care trends before, during and after the COVID-19 pandemic.
- Examining multiple gynaecological care pathways offers a detailed understanding of how different clinical contexts were affected by the pandemic.
- The observational nature of the study limits our ability to infer causality between the pandemic and the changes in care utilization.

For peer review only

For peer review only - http://bmjopen.bmj.com/site/about/guidelines.xhtml

INTRODUCTION

Healthcare systems are under increasing strain due to rising expenditures and limited resources.[1, 2] This results in a need for more sustainable and efficient care models that is increasing over time. Substitution of high intensity interventions with less resource-intensive alternatives may help if they result in adequate outcomes.[3] However, substituting high-intensity interventions, such as surgical procedures, with suitable less resource-intensive alternatives is often met with resistance due to clinical efficacy concerns, and organizational barriers to implementing such changes.[4]

The COVID-19 pandemic, with its widespread disruption of elective surgical procedures and resource allocation toward urgent and critical care, created a natural experiment to examine whether substitution increased in times of acute and prolonged scarcity.[5-8] The initial COVID-19 period showed a reduction of 37% of all healthcare services across 20 countries.[9] Non-COVID hospital admissions were reduced by 28%. A Dutch study by De Graaff et al. Showed that that mainly non-cancer surgical treatments were cancelled during months with high COVID-19 rates.[10] The combination of a sharp reduction in surgical capacity and a lacking perspective to recover delayed care created a window of opportunity for less capacity consuming, less invasive (non-)surgical alternatives. While most research to date study the impact of the reduction of surgical care, none describe the effect of the pandemic on substitution.[9, 11-14]

Within gynaecology, elective surgical procedures were significantly curtailed during the COVID-19 pandemic due to the limited surgical capacity and resource allocation toward urgent and critical care.[15-18] In benign gynaecology, surgical interventions, such as hysterectomies, are used to treat conditions such as bleeding disorders and uterine

For peer review only - http://bmjopen.bmj.com/site/about/guidelines.xhtml

fibroids.[19] While these surgeries effectively halt disease progression and improve quality of life, less invasive treatments like hormonal therapies and outpatient procedures are underutilized as an alternative, leading to unnecessary invasive and costly care.[20] While shifts towards less invasive gynaecological care have long been called for, actual substitution rates remained low before the pandemic.[21-22]

Given the growing scarcity of healthcare resources globally [23], this study aims to describe the pandemic's impact on surgical procedures and non-surgical care in benign gynaecology in the Netherlands. We hypothesize that acute surgical capacity constraints of labour-intensive surgical procedures were substituted by more efficient non-surgical alternatives across the six chosen pathways during the initial COVID-19 period due to resource shortages and the need for alternative treatment strategies.[11, 24]

Our goal is to uncover whether, and to what extent, substitution occurred and was sustained during and after this capacity squeeze of the operation theatre for an extended period. In addition we will describe patterns of healthcare deferral and the extent to which it was forgone for each of the pathways.

For peer review only

For peer review only - http://bmjopen.bmj.com/site/about/guidelines.xhtml

METHODS

Study design

This quantitative longitudinal study describes the use of benign gynaecological care pathways in the Netherlands before, during and after the COVID-19 pandemic. A care pathway encompasses a disease and all associated care products and activities, providing a comprehensive framework for managing the patient's entire course of care. We compared the initial pandemic year (2020) to four pre-pandemic years (2016–2019), as well as a late pandemic (2021) and a post-pandemic (2022) year.

The study focuses on six benign gynaecological pathways, classified using Dutch Diagnosis Treatment Combinations (DTCs): heavy menstrual blood loss (G11), uterine fibroids (G15), endometriosis (G17), prolapse (G25), infertility treatment (F11), and first trimester pregnancy complications (Z12). These care pathways are all part of the benign gynaecological diseases and contain elective surgical procedures as treatment options. These specific pathways were chosen because they offer one or several (non-)surgical alternatives with comparable outcomes, which made it possible for substitution of elective procedures to occur in the COVID-19 period.[25-31]

Data collection

Healthcare delivery across the six benign gynaecological pathways from 2016 to 2022 was analysed using Vektis and Dutch Hospital Data (DHD), accessed through Statistics Netherlands (CBS). Vektis is the Dutch healthcare information centre that collects, analyses, and provides data related to health insurance claims in the Netherlands. Its goal is to enhance transparency and improve the efficiency and quality of healthcare by offering insights based on claims data from health insurers. Dutch Hospital Data (DHD) is a central

For peer review only - http://bmjopen.bmj.com/site/about/guidelines.xhtml

organization in the Netherlands that collects, manages, and provides hospital data for analysis and research purposes. The DHD dataset includes data on patient admissions, diagnoses, procedures, and other care activities, derived from hospitals' electronic health records (EHRs). It is used to analyse healthcare utilization, quality, and efficiency within Dutch hospitals. Statistics Netherlands (Centraal Bureau voor de Statistiek, CBS) is the national statistical office that provides comprehensive data on a wide range of topics, including healthcare. The CBS dataset includes demographic, economic, and health-related data, including hospital admissions, mortality, healthcare expenditures, and population trends. CBS data often integrates with Vektis and DHD for broader analyses, offering insights into healthcare system performance and population health.

Pathway identification was based on Diagnosis Treatment Combinations (DTCs). Both surgical and non-surgical procedures were identified through healthcare products and activity numbers linked to individual patient records. The categorization of procedures as surgical or non-surgical was determined by these healthcare products and activity numbers. Surgical procedures are defined as procedures that require an anaesthesiologist present, typically in the operation room. Non-surgical procedures include less invasive alternatives that do not (see table 1 for distribution of surgical vs. non-surgical procedures and activities). A procedure was assigned to either the surgical- or non-surgical group if both healthcare products and activity numbers belonged to the same group. Records were excluded if the care product and activity numbers did not consistently align with either the surgical or non-surgical care group. The introduction date of new DTCs served as the marker for the commencement of surgical or non-surgical care.

For peer review only

For peer review only - http://bmjopen.bmj.com/site/about/guidelines.xhtml

Table 1. Distribution of procedures in surgical- or non-surgical care

Care pathway	Surgical	Non-surgical
Heavy menstrual bleeding	Hysterectomy (open/laparoscopic/vaginal/robot)	Endometrial Ablation<br>GNRH analogists<br>Hormonal Intra-Uterine Device<br>Oral Anticonception<br>NSAIDs<br>Tranexamic acid<br>Wait-and-see
Uterine fibroids	Hysterectomy (open/laparoscopic/vaginal/robot)<br>Myomectomy<br>Trans Cervical Removal of Myoma (TCRM)	Uterine Artery Embolization<br>GNRH analogists<br>Hormonal Intra-Uterine Device<br>Oral Anticonception<br>NSAIDs<br>Tranexamic acid<br>Wait-and-see
Endometriosis	Laparoscopic or laparotomic debulking of endometriosis	Hormonal Intra-Uterine Device<br>Oral Contraception<br>Wait-and-see
Prolapse	Prolapse surgery	Pelvic physiotherapy<br>Pessaries<br>Wait-and-see
First trimester pregnancy complications	Curettage<br>Laparoscopic or laparotomic ectopic pregnancy surgery	Misoprostol<br>Methotrexate treatment<br>Wait-and-see
Infertility treatment	Diagnostic/therapeutic laparoscopic surgery	Intra Uterine Insemination<br>In Vitro Fertilization<br>Wait-and-see

For peer review only - http://bmjopen.bmj.com/site/about/guidelines.xhtml

Population

Patients who received care within one of the specified gynaecological care pathways from January 1, 2016, to December 31, 2022, were included. Care contains the overall healthcare products, whether they are part of a DTC or labelled as 'other' and are given a start- and end date. Exclusions were made for patients under 18 years old and, specifically within the menstrual disorder pathway, for those over 51 years old to exclude most postmenopausal blood loss cases where no alternative treatment applies.

Dataset processing

To create our dataset, we had to combine several datasets from both DHD and Vektis within the Statistics Netherlands microenvironment. Combining, processing and cleaning of the datasets is done using RStudio. Combination of different datasets is done using individual anonymous patient numbers which overlap in the various datasets. We combined datasets to create a dataset containing rows of individual patient records, with columns containing: DTC, care product, care activity, care start- and end date, age. We used the abovementioned inclusion and exclusion criteria to filter within our dataset. Our dataset includes all Dutch patient records of the six care pathways over the years 2016 to 2022.

Outcome measures

Healthcare use during the pandemic year (2020) was compared to levels and trends from 2016–2019, 2021 and 2022. To account for seasonal variation and for graphical presentation only, a 3-month moving average was used to monthly absolute patient counts. Each point in the moving average represents the average of one preceding, the current and a subsequent month, to reduce noise. A predicted trendline for the years 2020 to 2022 was estimated based on the average annual growth from 2016 to 2019 combined with the 3-month moving

For peer review only - http://bmjopen.bmj.com/site/about/guidelines.xhtml

average of 2019 patient counts. This predicted trendline serves as a counterfactual to compare the years with COVID-19 to a fictional situation without COVID-19. The primary outcome was the trend in the total number of patients in both surgical and non-surgical procedure groups across the cohort periods. Secondary outcomes included trends within the individual pathways.

Statistical Analysis

Statistical analyses were conducted to test the significance of observed differences in descriptive data across years. Linear regressions were used to identify trends over time, with statistical significance set at $p < 0.05$. In the linear regression model, data from 2016 were used as the reference year. The year 2020 was included as a dummy variable to account for the impact of the COVID-19 pandemic. Additionally, a dummy variable for surgical care was included to differentiate between operative and non-operative care within the model. This allowed us to examine the effects of both the COVID-19 pandemic and the type of care on the outcomes of interest. No data imputation was performed for missing values. Data were analysed using RStudio (Version 4.3.0).

Patient and public involvement

There was no patient or public involvement in the design, conduct, reporting, or dissemination of this research. The study was conducted solely by the research team, and there were no consultations with patients or members of the public during its planning or execution.

For peer review only

For peer review only - http://bmjopen.bmj.com/site/about/guidelines.xhtml

RESULTS

A total of 2,281,613 patient records across six care pathways were included for the years 2016 to 2022 in the Netherlands. Table 2 shows the number of new records and the proportion of surgical care for each pathway over this period.

Table 2. Baseline characteristics care pathways per year

Care pathway	2016	2017	2018	2019	2020	2021	2022
Menstrual disorders (n)	89199	89942	94360	95111	88016	94006	92762
Surgical care (%)	14.2	14.3	14.0	13.2	11.4	11.6	12.0
Uterine fibroids (n)	22350	22924	23993	23581	21776	23148	24078
Surgical care (%)	17.9	17.3	17.1	17.2	16.2	16.6	16.5
Endometriosis (n)	16177	17291	19456	21692	22592	26532	29494
Surgical care (%)	8.7	8.5	8.7	8.8	7.0	6.8	7.4
Prolapse (n)	92152	92536	95888	97799	84956	92299	100561
Surgical care (%)	12.2	12.1	12.2	11.9	9.2	9.7	11.5
First trimester pregnancy complications (n)	39395	36248	37129	35809	34908	35323	33749
Surgical care (%)	30.0	26.7	24.5	24.0	21.8	20.4	20.2
Infertility treatment (n)	60884	60562	60806	60583	57383	59801	54362
Surgical care (%)	2.5	2.4	2.0	1.9	1.5	1.5	1.5
**TOTAL (N)**	320157	319503	331632	334575	309631	331109	335006
Surgical care (%)	13.3	12.7	12.4	11.9	10.1	10.1	10.9

■ = Covid-19 period

Overall, patient records increased from 320,157 in 2016 to 335,006 in 2022, representing a 4.6% rise. The highest number of records was in 2022 (335,006), while the lowest was in 2020 (309,631). The prolapse pathway had the largest volume, while endometriosis had the smallest. In 2020, the proportion of surgical care records declined across all pathways

For peer review only - http://bmjopen.bmj.com/site/about/guidelines.xhtml

compared to 2019. The greatest decrease was in the prolapse pathway (-2.7%), while the smallest was in the infertility treatment pathway (-0.4%).

Figure 1 illustrates total patient records for surgical and non-surgical care from 2016 to 2022, with a predicted trendline from 2020 to 2022.

The predicted trendlines reveal a decrease in actual patient numbers compared to predicted patient numbers for both surgical- and non-surgical procedures in the year 2020 compared to pre-COVID trends. The drop in 2020 amounts to a reduction of 6% (*P*=0.02) for non-surgical care group patient numbers and a reduction of 18.3% for surgical care (*P*<0.01 and compared to non-surgical care *P*=0.01). In the non-surgical care group patient numbers were restored to predicted levels by July 2020. In the surgical care group numbers remained below predicted trends throughout 2020 and 2021 and returned to the predicted trendline in 2022. A seasonal fluctuation is evident in the surgical care group over the years.

Figure 2 present graphs of the six care pathways separately. The graphs show the number of patient records for both surgical- and nonsurgical care from 2016 to 2022, with a predicted trendline from 2020 to 2022.

A similar trend pattern was observed across the care pathways for heavy menstrual blood loss, endometriosis, uterine fibroids, and prolapse. In these pathways, both surgical and non-surgical procedures declined during the COVID-19 period in 2020. Over time, surgical care did not return to pre-COVID trends, whereas non-surgical care did. In the care pathway heavy menstrual blood loss, the surgical procedures declined with 17.5% (*P*<0.01 and compared to non-surgical care *P*=0.009) while non-surgical procedures declined with 5.5%

For peer review only - http://bmjopen.bmj.com/site/about/guidelines.xhtml

For peer review only

(*P*=0.09). In the uterine fibroids care pathway surgical care was reduced by 12.6% (*P*<0.001 and compared to non-surgical care *P*=0.2) and nonsurgical care was reduced by 7.5% (*P*=0.01). Both types of care in the endometriosis care pathway dropped in amount. Surgical care dropped with 16.9% (*P*=0.1 and compared to non-surgical care *P*=0.7) and non-surgical care dropped with 5.4% (*P*=0.8). Most decline is seen in the care pathway prolapse, with a decline of 10.4% (*P*=0.7) of non-surgical procedures and of 17.5% (*P*=0.1 and compared to non-surgical care *P*=0.8) of surgical procedures.

The pathways for first trimester pregnancy complications and infertility treatment exhibited a pre-COVID pattern of declining surgical procedures and stable non-surgical procedures. During the initial COVID-19 period, both types of care declined, but they returned to pre-COVID trends by around June/July 2020. The first trimester pregnancy complications care pathway shows a decline of only 0.6% (*P*=1) of non-surgical procedures over 2020 compared to predicted trends, while the surgical procedures declined with 17.7% (*P*=0.1 and compared to non-surgical care *P*=0.6). Infertility treatment shows a similar pattern with a decline of 2.1% (*P*=0.6) of non-surgical procedures and of 19.3% (*P*<0.001 and compared to non-surgical care *P*=0.005) of surgical procedures.

For peer review only - http://bmjopen.bmj.com/site/about/guidelines.xhtml

DISCUSSION

In this quantitative cohort study, we evaluated the potential substitution of surgical procedures with non-surgical procedures across six gynaecological care pathways during the initial phase of the COVID-19 pandemic in the Netherlands. Our analysis revealed a significant reduction of benign gynaecological care of 18.3% for surgical and 6% for non-surgical procedures across all pathways over the whole of 2020. While non-surgical procedures resumed pre-pandemic trends by mid-2020, surgical procedures did not return to baseline levels. This implies that reductions in healthcare in 2020 were not compensated during the duration of the study. This trend was most pronounced in the prolapse pathway and was also observed in the pathways for menstrual disorders, uterine fibroids, and endometriosis.

In contrast, the care pathways for urgent first trimester pregnancy complications and infertility treatment showed pre-pandemic trends of declining surgical procedures and stable non-surgical procedures. In the case of first trimester pregnancy complications, there was minimal reduction in both surgical and non-surgical interventions in 2020. This may well result from the urgent character of this care pathway and is similar to the patterns for acute care during the pandemic.[10, 32, 33] Infertility care demonstrated a decrease in patient records during the beginning of the pandemic, with both procedural types returning to pre-pandemic patterns by mid-2020.

Our findings regarding the reduction in surgical procedures are consistent with existing literature documenting widespread disruptions in elective surgical care during the pandemic. Previous studies have highlighted similar reductions in elective surgery volumes, attributed to the redeployment of healthcare resources towards COVID-19 care and the imposition of

For peer review only - http://bmjopen.bmj.com/site/about/guidelines.xhtml

restrictions on elective procedures.[34] For example, a study in Japan reported an 8.5% decline in benign gynaecological surgeries in 2020 compared to 2019 [17], while New South Wales experienced a 37% reduction in general elective surgeries.[32]

Contrary to our initial hypothesis, we did not find evidence for an increase in the substitution of surgical procedures with non-surgical alternatives. Instead, both surgical and non-surgical procedures declined across multiple care pathways. This aligns with earlier reports of a decrease in both non-COVID medical and surgical hospitalizations by 22% and 33%, respectively [35], suggesting a more generalized reduction in healthcare capacity during the pandemic. This overall decrease may have resulted from a combination of reduced patient demand and reduced healthcare capacity. Data from the Dutch Healthcare Authority and ZorgDomein showed that the number of referrals during the first covid wave was 12% lower than in 2019, with a marked decline in gynaecology referrals between March and May 2020, before returning to normal by June.[36] To our surprise no increase of referrals was observed to recover delayed care. This decline in referrals contributed to the observed reduction in both surgical and non-surgical procedures within the gynaecological care pathways [18], but does not explain the sustained effect of continuously lower surgical procedure rates.

Patient-related factors likely influenced the reduction in referrals during the pandemic. Older patients, at higher risk for severe COVID-19 outcomes [37], may have been more hesitant to seek care, which could explain the pronounced decline in the prolapse pathway, predominantly affecting older individuals. Symptom severity and urgency also played a role; public health campaigns emphasized reducing in-person visits [38-40], likely deterring patients from seeking care for non-urgent conditions such as menstrual disorders, fibroids,

For peer review only

endometriosis, and prolapse. Stay-at-home orders may have heightened symptom awareness but also reduced referrals, as patients tolerated symptoms more easily in home-based settings.

Care utilization did not rebound in 2021 or 2022, suggesting missed care was not "caught up." Some patients may have permanently deferred surgery, experienced symptom fluctuations, or faced factors like mortality in older prolapse patients or menopause in those with heavy menstrual blood loss. These dynamics underscore the complexity of post-pandemic care patterns, requiring further study to assess their long-term impact on gynaecological practice.

Gynaecologists' perception of non-surgical alternatives as less effective than surgical procedures may have contributed to deferred treatments. For instance, uterine-preserving options like uterine artery embolization may be viewed as less reliable than hysterectomy, leading to delays until surgical intervention became feasible.[41] Previous research indicates that not all gynaecologists discuss uterine embolization as a treatment for fibroids.[42] When surgical procedures are considered the gold standard, particularly for conditions like prolapse, fibroids, or advanced endometriosis, gynaecologists may recommend delaying treatment rather than pursuing less effective non-surgical alternatives.

Deferring surgery may be inappropriate for moderate to severe cases like prolapse or endometriosis, where symptom progression is expected. According to the Dutch association of medical specialists (2023)[43], patient preferences also play a role, as some patients may opt to wait for definitive surgery after failed conservative treatments or view non-surgical options as temporary, leading to prioritization of surgical care despite pandemic resource constraints.

For peer review only - http://bmjopen.bmj.com/site/about/guidelines.xhtml

Pandemic-related staff redeployment, including anaesthesiologists to COVID-19 units, reduced operating room capacity for elective procedures.[5] Gynaecological procedures like those for menstrual disorders, fibroids, and prolapse were deferred, while urgent surgeries, such as those for first-trimester pregnancy complications, continued due to their non-elective nature. Healthcare staff shortages from COVID-19 infections further delayed elective surgeries and outpatient intakes.[44] Combined with patient reluctance and perceptions of non-surgical alternatives, these factors likely drove the significant procedural volume reductions observed.

Strengths and limitations

A major strength of our study is the large dataset spanning all benign gynaecological elective care by clinics, hospitals and academic hospitals in all of The Netherlands for a period of six years. This allows a detailed longitudinal insight into gynaecological care trends before, during and after the COVID-19 pandemic. Moreover, the Dutch Hospital Data (DHD) provider follows standardized data collection protocols that enhance the comparability of our findings. In addition, by examining multiple gynaecological care pathways, ranging from menstrual disorders to prolapse, our study offers a detailed understanding of how different clinical contexts were affected by the pandemic.

Nevertheless, there are important limitations. The observational nature of the study limits our ability to infer causality between the pandemic and the changes in care utilization. Additionally, we did not assess changes in primary health care use, which might have substituted a share of the secondary care, and patient outcomes, which restricts our ability to evaluate the effectiveness of the reduced or substituted non-surgical procedures. This

For peer review only - http://bmjopen.bmj.com/site/about/guidelines.xhtml

latter limitation is particularly relevant when considering the long-term consequences of deferred surgical interventions.

Implications for practice

Our findings have significant implications for clinical practice and healthcare policy. The observed reduction in both surgical and non-surgical procedures during the initial phase of the COVID-19 pandemic, with no evidence of subsequent compensation, suggests a fundamental shift in healthcare delivery: care was not deferred or substituted, but it appears to have vanished. This phenomenon of forgone care necessitates a deeper understanding of the underlying causes and the potential long-term consequences for patients and healthcare systems.

First, it is critical to investigate what happened to the patients who did not receive care during this period. Did they seek alternative forms of care, such as treatment in primary care settings or through conservative management approaches? Or did they forego care altogether, potentially leading to unresolved health issues or adverse outcomes? Understanding these dynamics is essential to ascertain whether this reduction in care resulted in unmet medical needs or whether it represents an unintentional shift toward more appropriate care delivery. Such insights could provide valuable context for assessing whether these changes align with principles of necessity and proportionality in healthcare provision.

Moreover, the long-term effects of this vanished care must be studied in detail. Questions remain as to whether the reduced volume of both surgical and non-surgical procedures reflects changes on the demand side during crisis, e.g. in disease incidence, patient preferences, or systemic barriers to access. Alternatively, the crisis might have affected the

For peer review only - http://bmjopen.bmj.com/site/about/guidelines.xhtml

use of care through supply side changes. Was the decline driven by restricted health care capacity: resource limitations, workforce shortages, or a broader recalibration of clinical priorities during the pandemic? Understanding these drivers will inform strategies to balance immediate care demands with the need to ensure equitable access in future crises.

A better understanding might also help to define the appropriateness of care in non-crisis settings. The COVID-19 pandemic created a unique opportunity to critically evaluate which procedures are essential and which may be of limited benefit. However, the observed reduction in care must not be universally interpreted as progress towards more appropriate care.

For peer review only

CONCLUSION

The COVID-19 pandemic significantly disrupted both surgical and non-surgical procedures within benign gynaecology pathways. Reduced uptake of care during the COVID waves was not recovered but was forgone. The reduction in surgical procedures did not correspond with increased use of non-surgical alternatives. This raises important societal questions about consequences of unused care and especially whether the reduction in surgical procedures is desirable or has left patients with a disproportionately reduced quality of life.

Future research should prioritize evaluating the impact of this disruption at both the patient and the societal level. Qualitative research is needed to understand the individual patient experiences and outcomes, while quantitative studies should assess the broader implications for care pathways and healthcare costs. Our findings offer valuable insights that can guide healthcare policy and practice, emphasizing the importance of developing resilient systems capable of delivering high-quality care in the face of future disruptions and relative scarcity of medical capacity.

For peer review only

For peer review only - http://bmjopen.bmj.com/site/about/guidelines.xhtml

Data sharing statement

The data used for this study were obtained from Statistics Netherlands (Centraal Bureau voor de Statistiek, CBS) under a data-sharing agreement. Due to privacy regulations and the confidentiality of individual-level data, the dataset cannot be shared publicly. Access to CBS data is restricted and requires approval through their secure microdata services, available to registered institutions under specific conditions.

Authorship statement

All authors made substantial contributions to the conception, design, and acquisition of data for the work, as well as the analysis and interpretation of the data. E.L.M. Velthuijs and I. Ismail had a particularly significant role in the analysis of the data. E.L.M. Velthuijs drafted the manuscript, with critical revisions from I. Ismail, A.H.E. Koolman, R.A. de Leeuw, and W.J.K. Hehenkamp. All authors reviewed the manuscript critically for important intellectual content. Each author gave final approval of the version to be published and agrees to be accountable for all aspects of the work, ensuring that questions related to the accuracy or integrity of any part of the work are appropriately investigated and resolved.

Competing interest statement

None of the authors have a potential conflict of interest.

Funding

This study was funded by ZonMw, the Dutch Organization for Health Research and Development, under project number 05160482240003.

For peer review only

REFERENCES

1. OECD. Ready for the Next Crisis? Investing in Health System Resilience. 2023.

2. Cynthia Cox JO, Emma Wager, Krutika Amin. Health Care Costs and Affordability. KFF; 2024.

3. Adan I, Bekkers J, Dellaert N, et al. Improving operational effectiveness of tactical master plans for emergency and elective patients under stochastic demand and capacitated resources. European Journal of Operational Research. 2011;213(1):290-308.

4. Cowie J, Nicoll A, Dimova ED, et al. The barriers and facilitators influencing the sustainability of hospital-based interventions: a systematic review. BMC Health Services Research. 2020;20(1):588.

5. CMS. Adult Elective Surgery and Procedures Recommendations:

Limit all non-essential planned surgeries and procedures, including dental, until further notice. 2020.

6. Francis N, Dort J, Cho E, et al. SAGES and EAES recommendations for minimally invasive surgery during COVID-19 pandemic. Surgical Endoscopy. 2020;34(6):2327-31.

7. Pryor A. SAGES and EAES Recommendations Regarding Surgical Response to COVID-19 Crisis 2020 [Available from: https://www.sages.org/recommendations-surgical-response-covid-19/.

8. COVIDSurgCollaborative. Global guidance for surgical care during the COVID-19 pandemic. British Journal of Surgery. 2020;107(9):1097-103.

For peer review only

For peer review only - http://bmjopen.bmj.com/site/about/guidelines.xhtml

9. Moynihan R, Sanders S, Michaleff ZA, et al. Impact of COVID-19 pandemic on utilisation of healthcare services: a systematic review. BMJ Open. 2021;11(3):e045343.

10. de Graaff MR, Hogenbirk RNM, Janssen YF, et al. Impact of the COVID-19 pandemic on surgical care in the Netherlands. Br J Surg. 2022;109(12):1282-92.

11. Winkelmann J, Webb E, Williams GA, et al. European countries' responses in ensuring sufficient physical infrastructure and workforce capacity during the first COVID-19 wave. Health Policy. 2022;126(5):362-72.

12. Moynihan R, Johansson M, Maybee A, et al. Covid-19: an opportunity to reduce unnecessary healthcare. BMJ. 2020;370:m2752.

13. Oosterhoff M, Kouwenberg LHJA, Rotteveel AH, et al. Estimating the health impact of delayed elective care during the COVID -19 pandemic in the Netherlands. Social Science & Medicine. 2023;320:115658.

14. van Giessen A, de Wit A, van den Brink C, et al. Impact van de eerste COVID-19 golf op de reguliere zorg en gezondheid : Inventarisatie van de omvang van het probleem en eerste schatting van gezondheidseffecten. Impact of the first wave of COVID-19 on regular healthcare and health : Assessment of the extent of the problem and first estimation of the health effects: Rijksinstituut voor Volksgezondheid en Milieu RIVM; 2020.

15. Diaz A, Sarac BA, Schoenbrunner AR, et al. Elective surgery in the time of COVID-19. The American Journal of Surgery. 2020;219(6):900-2.

16. Spinelli A, Pellino G. COVID-19 pandemic: perspectives on an unfolding crisis. British Journal of Surgery. 2020;107(7):785-7.

For peer review only - http://bmjopen.bmj.com/site/about/guidelines.xhtml

17. Komatsu H, Nakagawa S, Yamagami W, et al. Impact of the COVID-19 pandemic on surgery for benign diseases in gynecology: A nationwide survey by the Japan Society of Obstetrics and Gynecology. Journal of Obstetrics and Gynaecology Research. 2023;49(1):314-20.

18. Nederlandse Zorgautoriteit. Analyse van de gevolgen van de coronacrisis voor de reguliere ziekenhuiszorg - 26 mei 2020. 2020.

19. Rahman R, Gupta S, Manyonda I. Hysterectomy for benign gynaecological disease. Obstetrics, Gynaecology & Reproductive Medicine. 2017;27(4):125-31.

20. Vink Maarten D.H.P., France R.M., van Wezep Tim C., et al. Practice variation in the stepped care approach to idiopathic heavy menstrual bleeding: A population-based study. European Journal of Obstetrics & Gynecology and Reproductive Biology; 2023. p. 6-12.

21. FederatieMedischSpecialisten. Handreiking Implementatie Passende zorg Bouwstenen voor de praktijk. demedischspecialist.nl2023.

22. Cohen ES, Kouwenberg LHJA, Moody KS, et al. Environmental sustainability in obstetrics and gynaecology: A systematic review. BJOG: An International Journal of Obstetrics & Gynaecology. 2024;131(5):555-67.

23. WorldHealthOrganizationSecretariat. Global strategy on human resources for health: Workforce 2030. Geneva: WHO; 2016.

24. Burki T. Global shortage of personal protective equipment. The Lancet Infectious Diseases. 2020;20(7):785-6.

For peer review only

For peer review only - http://bmjopen.bmj.com/site/about/guidelines.xhtml

25. Abdool Z, Thakar R, Sultan AH, et al. Prospective evaluation of outcome of vaginal pessaries versus surgery in women with symptomatic pelvic organ prolapse. International Urogynecology Journal. 2011;22(3):273-8.

26. Brennan A, Hickey M. Abnormal uterine bleeding: managing endometrial dysfunction and leiomyomas. Medical Journal of Australia. 2018;208(2):90-5.

27. Kalaitzopoulos DR, Samartzis N, Kolovos GN, et al. Treatment of endometriosis: a review with comparison of 8 guidelines. BMC Women's Health. 2021;21(1):397.

28. Matteson KA, Abed H, Wheeler TL, et al. A systematic review comparing hysterectomy with less-invasive treatments for abnormal uterine bleeding. J Minim Invasive Gynecol. 2012;19(1):13-28.

29. Rezende GP, Venturini MC, Kawagoe LN, et al. Surgery vs. hormone-based treatment for pain control in deep infiltrating endometriosis: a retrospective cohort study. Curr Med Res Opin. 2022;38(4):641-7.

30. van der Kooij SM, Bipat S, Hehenkamp WJK, et al. Uterine artery embolization versus surgery in the treatment of symptomatic fibroids: a systematic review and metaanalysis. American Journal of Obstetrics and Gynecology. 2011;205(4):317.e1-.e18.

31. van der Vaart LR, Vollebregt A, Milani AL, et al. Pessary or surgery for a symptomatic pelvic organ prolapse: the PEOPLE study, a multicentre prospective cohort study. Bjog. 2022;129(5):820-9.

32. McBride KE, Steffens D, Solomon MJ. From the sidelines: The indirect repercussions of COVID-19 on the delivery of hospital surgical services. ANZ J Surg. 2021;91(7-8):1345-51.

For peer review only - http://bmjopen.bmj.com/site/about/guidelines.xhtml

33. Nederlandse Zorgautoriteit. Analyse van de gevolgen van de coronacrisis voor de reguliere zorg 2020.

34. Mayol J, Fernández Pérez C. Elective surgery after the pandemic: waves beyond the horizon. British Journal of Surgery. 2020;107(9):1091-3.

35. Gasch-Illescas A, Calle-Serrano M, Vallejo-Vaz AJ, et al. Impact of the first wave of the COVID-19 pandemic on non-COVID inpatient care in southern Spain. Sci Rep. 2023;13(1):1634.

36. Zorgdomein. Uitgestelde zorg loopt in 2020 op tot ruim 1,7 miljoen verwijzingen2020. Available from: https://zorgdomein.com/nieuws/uitgestelde-zorg-loopt-in-2020-op-tot-ruim-17-miljoen-verwijzingen/.

37. CDC. Underlying Medical Conditions Associated with Higher Risk for Severe COVID-19: Information for Healthcare Professionals 2021 [updated 24-4-2024. Available from: https://www.cdc.gov/coronavirus/2019-ncov/hcp/clinical-care/underlyingconditions.html.

38. Dupraz J, Le Pogam M-A, Peytremann-Bridevaux I. Early impact of the COVID-19 pandemic on in-person outpatient care utilisation: a rapid review. BMJ Open. 2022;12(3):e056086.

39. Green MA, McKee M, Katikireddi SV. Remote general practitioner consultations during COVID-19. The Lancet Digital Health. 2022;4(1):e7.

40. Jaffe TA, Hayden E, Uscher-Pines L, et al. Telehealth use in emergency care during coronavirus disease 2019: a systematic review. Journal of the American College of Emergency Physicians Open. 2021;2(3):e12443.

For peer review only

For peer review only - http://bmjopen.bmj.com/site/about/guidelines.xhtml

41. Gupta JK, Sinha A, Lumsden MA, et al. Uterine artery embolization for symptomatic uterine fibroids. Cochrane Database Syst Rev. 2012(5):Cd005073.

42. de Bruijn AM, Huisman J, Hehenkamp WJK, et al. Implementation of uterine artery embolization for symptomatic fibroids in the Netherlands: an inventory and preference study. CVIR Endovasc. 2019;2(1):18.

43. FederatieMedischSpecialisten. Richtlijn Hevig menstrueel bloedverlies (HMB). Richtlijnendatabase; 2023.

44. Nederlandse Zorgautoriteit. Monitor Toegankelijkheid van Zorg; gevolgen van Covid-19 - 26 augustus 2021. 2021.

For peer review only

For peer review only - http://bmjopen.bmj.com/site/about/guidelines.xhtml

Figure 1. Total patient count surgical- and nonsurgical care timeline 2016-2022 for the 6 care pathways combined [a]

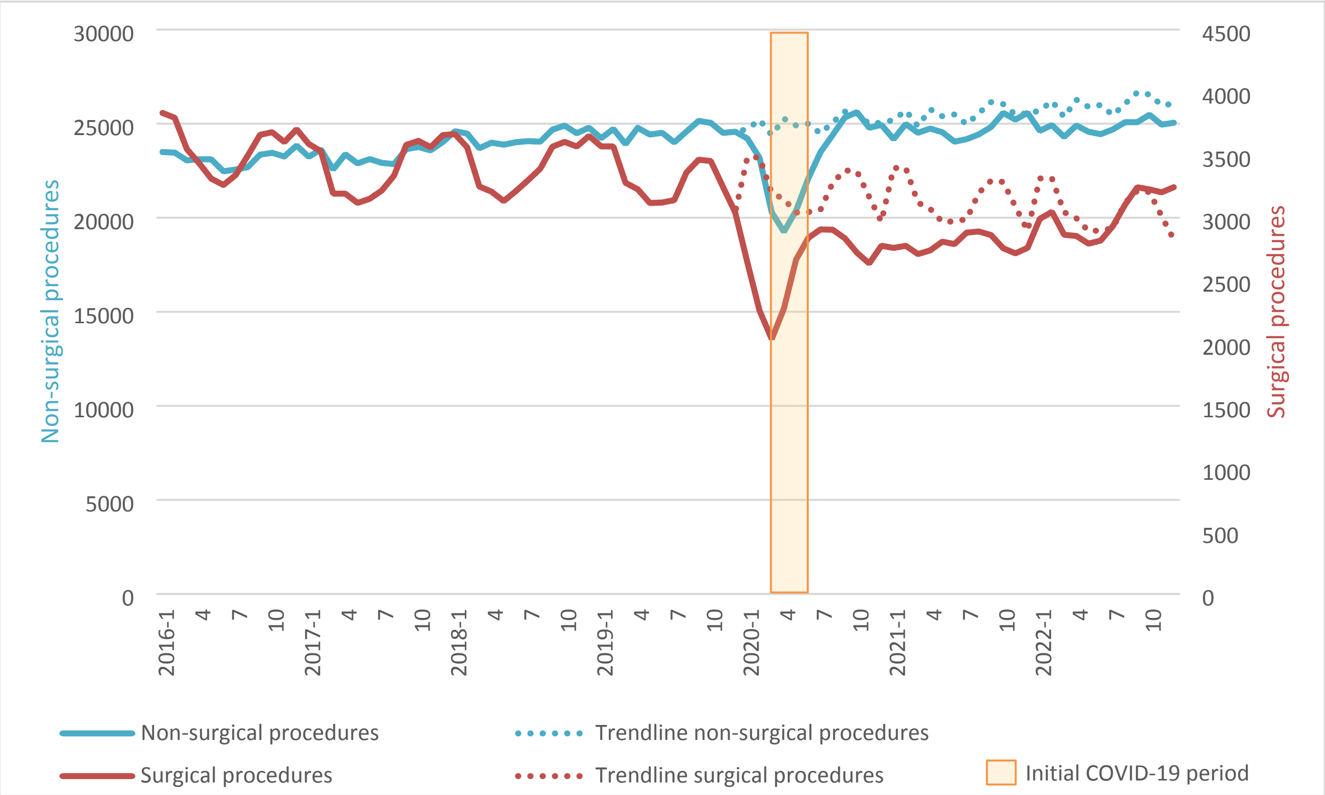

[a] Trendlines are presented using three-months moving averages

review only

Figure 2. Six care pathways surgical- and nonsurgical care timeline 2016-2022 [a]

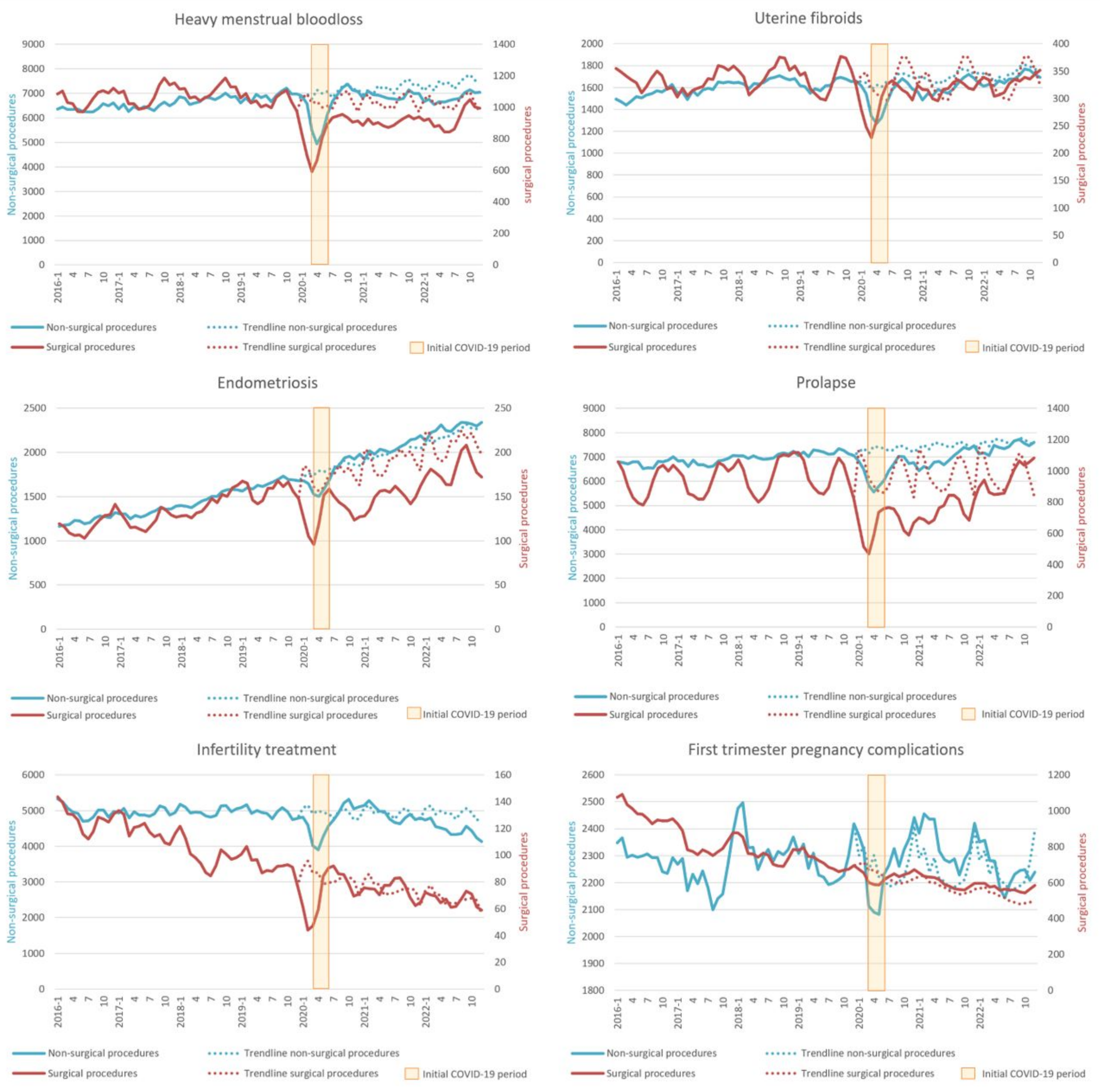

[a] Trendlines are presented using three-months moving averages

Substitution of Surgical Care within Benign Gynaecology during Covid-19: Waste of a Good Crisis? – A quantitative longitudinal study in the Netherlands

Journal:	*BMJ Open*
Manuscript ID	bmjopen-2025-100357.R1
Article Type:	Original research
Date Submitted by the Author:	19-Jun-2025
Complete List of Authors:	Velthuijs, Eva; Amsterdam UMC Locatie AMC, Gynaecology & Obstetrics<br>Ismail, Ismail; Vrije Universiteit Amsterdam, Health economics<br>Koolman, Xander ; VU Amsterdam<br>de Leeuw, Robert ; Amsterdam UMC Locatie AMC, Department of Gynaecology and Obstetrics<br>Hehenkamp, Wouter; Amsterdam UMC Locatie AMC, Obstetrics and Gynaecology
**Primary Subject Heading**:	Obstetrics and gynaecology
Secondary Subject Heading:	Health economics, Surgery
Keywords:	COVID-19, Delivery of Health Care, Integrated, GYNAECOLOGY

SCHOLARONE™
Manuscripts

I, the Submitting Author has the right to grant and does grant on behalf of all authors of the Work (as defined in the below author licence), an exclusive licence and/or a non-exclusive licence for contributions from authors who are: i) UK Crown employees; ii) where BMJ has agreed a CC-BY licence shall apply, and/or iii) in accordance with the terms applicable for US Federal Government officers or employees acting as part of their official duties; on a worldwide, perpetual, irrevocable, royalty-free basis to BMJ Publishing Group Ltd ("BMJ") its licensees and where the relevant Journal is co-owned by BMJ to the co-owners of the Journal, to publish the Work in this journal and any other BMJ products and to exploit all rights, as set out in our licence.

The Submitting Author accepts and understands that any supply made under these terms is made by BMJ to the Submitting Author unless you are acting as an employee on behalf of your employer or a postgraduate student of an affiliated institution which is paying any applicable article publishing charge ("APC") for Open Access articles. Where the Submitting Author wishes to make the Work available on an Open Access basis (and intends to pay the relevant APC), the terms of reuse of such Open Access shall be governed by a Creative Commons licence – details of these licences and which Creative Commons licence will apply to this Work are set out in our licence referred to above.

Other than as permitted in any relevant BMJ Author's Self Archiving Policies, I confirm this Work has not been accepted for publication elsewhere, is not being considered for publication elsewhere and does not duplicate material already published. I confirm all authors consent to publication of this Work and authorise the granting of this licence.

For peer review only

Title

Substitution of Surgical Care within Benign Gynaecology during Covid-19: Waste of a Good Crisis? – A quantitative longitudinal study in the Netherlands

Authors

E.L.M. Velthuijs (MD)[1], I. Ismail (Msc)[2], A.H.E. Koolman (PhD)[2], R.A. de Leeuw (MD, PhD)[1], W.J.K. Hehenkamp (MD, PhD)[1]

1. Department of Obstetrics and Gynaecology, Amsterdam UMC, Location VU/AMC, Amsterdam, The Netherlands

2. Department of Health Economics, Vrije Universiteit, Amsterdam, The Netherlands

Corresponding author:

E.L.M. Velthuijs

Department of Obstetrics and Gynaecology, Amsterdam UMC, Meibergdreef 9, Amsterdam 1105 AZ, The Netherlands

Email: e.velthuijs@amsterdamumc.nl

Manuscript word count: 3482

For peer review only - http://bmjopen.bmj.com/site/about/guidelines.xhtml

KEY WORDS

COVID-19, delivery of healthcare, gynaecologic surgical procedure

For peer review only

For peer review only - http://bmjopen.bmj.com/site/about/guidelines.xhtml

ABSTRACT

Objective: To examine the impact of the COVID-19 pandemic on the substitution of surgical procedures in benign gynaecology in the Netherlands.

Design: Quantitative longitudinal study evaluating the effects of the COVID-19 pandemic.

Setting: Nationwide healthcare delivery was analysed across six benign gynaecological pathways from 2016 to 2022 using Vektis and Dutch Hospital Data (DHD), accessed via Statistics Netherlands (CBS).

Participants: The study focused on six benign gynaecological pathways classified using Dutch Diagnosis Treatment Combinations (DTCs): heavy menstrual blood loss (G11), uterine fibroids (G15), endometriosis (G17), prolapse (G25), infertility treatment (F11), and first trimester pregnancy complications (Z12). All patients receiving care within these pathways between January 1, 2016, and December 31, 2022, were included. Exclusions applied to all patients under 18 years old and, only within the menstrual disorder pathway, patients over 51 years old to exclude most postmenopausal blood loss cases where no alternative treatment applies.

Interventions: Cohorts from the initial pandemic year (2020) were compared to four pre-pandemic cohorts (2016–2019) and late-pandemic (2021) and post-pandemic (2022) cohorts.

Primary and secondary outcome measures: The primary outcome was the trend in the total number of patients in surgical and non-surgical procedure groups across cohort periods. Secondary outcomes included trends within individual pathways.

Results: The analysis identified a significant reduction in benign gynaecological care during 2020, with an 18.3% (P<0.001) decrease in surgical procedures and a 6% (P=0.02) decrease in non-surgical procedures across all pathways. During COVID-19, surgical care showed the

For peer review only - http://bmjopen.bmj.com/site/about/guidelines.xhtml

largest significant declines in infertility treatment (−19.3%, P<0.001) and prolapse and heavy menstrual bleeding (both −17.5%, P<0.001), while non-surgical care decreased less markedly, with the largest significant drop in prolapse care (−10.4%, P=0.001). Non-surgical procedures generally returned to pre-pandemic levels, whereas surgical care remained reduced. These differences were conducted using regression models that adjusted for time trends and the COVID-related impact.

Conclusions: The COVID-19 pandemic significantly disrupted both surgical and non-surgical procedures within benign gynaecological pathways. Reduced care uptake during the pandemic waves was not recovered but instead forgone. The reduction in surgical procedures did not correspond with increased use of non-surgical alternatives. Future research should prioritize evaluating the long-term impacts of this disruption on patients and society.

Trial Registration: Not applicable.

For peer review only

STRENGTHS AND LIMITATIONS OF THIS STUDY

- The large dataset spanning allows a detailed longitudinal insight into gynaecological care trends before, during and after the COVID-19 pandemic.
- Examining multiple gynaecological care pathways offers a detailed understanding of how different clinical contexts were affected by the pandemic.
- The observational nature of the study limits our ability to infer causality between the pandemic and the changes in care utilization.

For peer review only

INTRODUCTION

Healthcare systems are under increasing strain due to rising expenditures and limited resources.[1, 2] This results in a need for more sustainable and efficient care models that is increasing over time. Substitution of high intensity interventions, such as surgical procedures, with less resource-intensive alternatives may help if they result in adequate outcomes.[3] However, such substitution is often hindered by concerns about clinical efficacy and organizational resistance.[4]

The COVID-19 pandemic, with its widespread disruption of elective surgical procedures and resource allocation toward urgent and critical care, created a natural experiment to examine whether substitution increased in times of acute and prolonged scarcity.[5-8] The initial COVID-19 period showed a reduction of 37% of all healthcare services across 20 countries.[9] Non-COVID hospital admissions were reduced by 28%. In the Netherlands, mainly non-cancer surgical treatments were cancelled during COVID-19 peaks.[10] National policies restricted non-urgent hospital care, especially during the first wave (March–May 2020), the second wave (October 2020–February 2021), and subsequent surges in 2021. Formal triage measures for surgical care, led by the Dutch Federation of Medical Specialists, were implemented to determine which procedures should be postponed.[11] Restrictions were lifted gradually during 2022 as hospital pressures subsided.The combination of a sharp reduction in surgical capacity and a lacking perspective to recover delayed care created a window of opportunity for less capacity consuming, less invasive (non-)surgical alternatives. While most research to date study the impact of the reduction of surgical care, none describe the effect of the pandemic on substitution.[9, 12-15]

For peer review only - http://bmjopen.bmj.com/site/about/guidelines.xhtml

Within gynaecology, elective surgical procedures were significantly curtailed during the COVID-19 pandemic due to the limited surgical capacity and resource allocation toward urgent and critical care.[16-19] In benign gynaecology, surgical interventions, such as hysterectomies, are used to treat conditions such as bleeding disorders and uterine fibroids.[20] While these surgeries effectively halt disease progression and improve quality of life, less invasive treatments like hormonal therapies and outpatient procedures are underutilized as an alternative, leading to unnecessary invasive and costly care.[21] While shifts towards less invasive gynaecological care have long been called for, actual substitution rates remained low before the pandemic.[22-23]

Given the growing scarcity of healthcare resources globally [24], this study aims to describe the pandemic's impact on surgical procedures and non-surgical care in benign gynaecology in the Netherlands. We hypothesize that acute surgical capacity constraints of labour-intensive surgical procedures were substituted by more efficient non-surgical alternatives across the six chosen pathways during the initial COVID-19 period due to resource shortages and the need for alternative treatment strategies.[12, 25]

Our goal is to uncover whether, and to what extent, substitution occurred and was sustained during and after this capacity squeeze of the operation theatre for an extended period. In addition we will describe patterns of healthcare deferral and the extent to which it was forgone for each of the pathways.

For peer review only - http://bmjopen.bmj.com/site/about/guidelines.xhtml

METHODS

Study design

This quantitative longitudinal study describes the use of benign gynaecological care pathways in the Netherlands before, during and after the COVID-19 pandemic. A care pathway encompasses a disease and all associated care products and activities, providing a comprehensive framework for managing the patient's entire course of care. We compared the initial pandemic year (2020) to four pre-pandemic years (2016–2019), as well as a late pandemic (2021) and a post-pandemic (2022) year.

The study focuses on six benign gynaecological pathways, classified using Dutch Diagnosis Treatment Combinations (DTCs): heavy menstrual bleeding (G11), uterine fibroids (G15), endometriosis (G17), prolapse (G25), infertility treatment (F11), and first trimester pregnancy complications (Z12). These conditions were selected because they include both elective surgical and non-surgical treatment options with comparable outcomes, allowing for potential substitution during periods of surgical capacity restriction.[26-32]

Data collection

Healthcare delivery across the six benign gynaecological pathways from 2016 to 2022 was analysed using Vektis and Dutch Hospital Data (DHD), accessed through Statistics Netherlands (CBS). Vektis is the Dutch healthcare information centre that collects and analyses health insurance claims to support transparency and improve healthcare quality and efficiency. Dutch Hospital Data (DHD) manages and provides hospital data derived from electronic health records (EHRs), including patient admissions, diagnoses, procedures, and other care activities. These datasets together provide near-complete coverage of hospital-based gynaecological care in the Netherlands. DHD captures virtually all hospital claims data,

For peer review only

while Vektis includes claims from all Dutch health insurers. Statistics Netherlands (Centraal Bureau voor de Statistiek, CBS), the national statistical office, provides comprehensive data on a wide range of topics, including healthcare. CBS data integrates with Vektis and DHD offering broader insights into healthcare performance and population health.

Pathway identification was based on Diagnosis Treatment Combinations (DTCs). Both surgical and non-surgical procedures were identified through healthcare products and activity numbers linked to individual patient records. The categorization of procedures as surgical or non-surgical was determined by these healthcare products and activity numbers. Surgical procedures are defined as procedures that require an anaesthesiologist present, typically in the operation room. Non-surgical procedures include less invasive alternatives that do not. Procedures were only included if both product and activity codes consistently belonged to the same category. Records with inconsistent classification were excluded.The introduction date of a new DTC marked the beginning of surgical or non-surgical care. Table 1 provides an overview of the classification and distribution of procedures within the selected DTCs.

Table 1. Distribution of procedures in surgical- or non-surgical care

Care pathway	Surgical	Non-surgical
Heavy menstrual bleeding	Hysterectomy (open/laparoscopic/vaginal/robot)	Endometrial Ablation<br>GNRH analogists<br>Hormonal Intra-Uterine Device<br>Oral Anticonception<br>NSAIDs<br>Tranexamic acid<br>Wait-and-see

For peer review only - http://bmjopen.bmj.com/site/about/guidelines.xhtml

Uterine fibroids	Hysterectomy (open/laparoscopic/vaginal/robot)<br><br>Myomectomy<br><br>Trans Cervical Removal of Myoma (TCRM)	Uterine Artery Embolization<br><br>GNRH analogists<br><br>Hormonal Intra-Uterine Device<br><br>Oral Anticonception<br><br>NSAIDs<br><br>Tranexamic acid<br><br>Wait-and-see
Endometriosis	Laparoscopic or laparotomic debulking of endometriosis	Hormonal Intra-Uterine Device<br><br>Oral Contraception<br><br>Wait-and-see
Prolapse	Prolapse surgery	Pelvic physiotherapy<br>Pessaries<br>Wait-and-see
First trimester pregnancy complications	Curettage<br><br>Laparoscopic or laparotomic ectopic pregnancy surgery	Misoprostol<br><br>Methotrexate treatment<br><br>Wait-and-see
Infertility treatment	Diagnostic/therapeutic laparoscopic surgery	Intra Uterine Insemination<br><br>In Vitro Fertilization<br><br>Wait-and-see

Population

Patients who received care within one of the specified gynaecological care pathways from January 1, 2016, to December 31, 2022, were included. Care contains the overall healthcare products, whether they are part of a DTC or labelled as 'other' and are given a start- and end date. Exclusions were made for patients under 18 years old and, specifically within the menstrual disorder pathway, for those over 51 years old to exclude most postmenopausal bleeding cases where no alternative treatment applies.

For peer review only - http://bmjopen.bmj.com/site/about/guidelines.xhtml

Dataset processing

To create our dataset, several DHD and Vektis sources were combined within the CBS microenvironment. Combining, processing and cleaning of the datasets is done using RStudio. Due to mandatory national reporting, data completeness was high, and no imputation was needed. Datasets were linked via anonymous patient identifiers shared across sources. The final dataset consisted of patient-level rows with columns for DTC, care product, care activity, care start- and end date, and age. Inclusion and exclusion criteria were applied accordingly. Our dataset includes all Dutch patients who received care within the six selected pathways between 2016 and 2022.

Outcome measures

Healthcare use during the pandemic year (2020) was compared to levels and trends from 2016–2019, 2021 and 2022. To account for seasonal variation and for graphical presentation only, a 3-month moving average was used to monthly absolute patient counts. Each point in the moving average represents the average of one preceding, the current and a subsequent month, to reduce noise. A predicted trendline for the years 2020 to 2022 was estimated to serve as a counterfactual, representing a scenario without COVID-19. This prediction was constructed by calculating the average annual growth rate in patient volumes between 2016 and 2019, and applying this rate to the baseline monthly volumes of 2019. This approach is an extrapolation model based solely on monthly data in patient volumes. The primary outcome was the trend in total patient numbers in surgical and non-surgical procedure groups across the cohort periods. Secondary outcomes included trends within individual pathways.

For peer review only - http://bmjopen.bmj.com/site/about/guidelines.xhtml

Statistical Analysis

Statistical analyses were conducted to test the significance of observed differences in descriptive data across years. To assess raw associations we used linear regression for continuous outcomes. To express regression outcomes in percentages, all continuous outcomes were log-transformed prior to analysis. When the distribution of the error term was not normal, we used non-parametric bootstrap to compute p-values. Where relevant, year-to-year differences in proportions were tested using chi-square tests. Statistical significance was set at $p < 0.05$. In the linear regression models, 2016 was used as the reference year. The year 2020 was included as a dummy variable to account for the impact of the COVID-19 pandemic. Additionally, a dummy variable for surgical care was included to differentiate between operative and non-operative care within the model. This enabled analysis of both pandemic and procedure type effects. Missing data were minimal and no imputation was performed. Data were analysed using RStudio (Version 4.3.0).

Patient and public involvement

There was no patient or public involvement in the design, conduct, reporting, or dissemination of this research. The study was conducted solely by the research team, and there were no consultations with patients or members of the public during its planning or execution.

For peer review only

RESULTS

A total of 2,281,613 patient records across six care pathways were included for the years 2016 to 2022 in the Netherlands. Table 2 shows the number of new records and the proportion of surgical care for each pathway over this period.

Table 2. Baseline characteristics care pathways per year

Care pathway	2016	2017	2018	2019	2020	2021	2022
Menstrual disorders (n)	89199	89942	94360	95111	88016	94006	92762
Surgical care (%)	14.2	14.3	14.0	13.2	11.4	11.6	12.0
Δ %[a] (*P*-value)		+0.1 (0.55)	-0.3 (0.07)	-0.8 (<0.001)	-1.8 (<0.001)	+0.2 (0.18)	+0.4 (0.01)
Uterine fibroids (n)	22350	22924	23993	23581	21776	23148	24078
Surgical care (%)	17.9	17.3	17.1	17.2	16.2	16.6	16.5
Δ %[a] (*P*-value)		-0.6 (0.1)	-0.2 (0.58)	+0.1 (0.78)	-1.0 (0.005)	+0.4 (0.26)	-0.1 (0.78)
Endometriosis (n)	16177	17291	19456	21692	22592	26532	29494
Surgical care (%)	8.7	8.5	8.7	8.8	7.0	6.8	7.4
Δ %[a] (*P*-value)		-0.2 (0.54)	+0.2 (0.51)	+0.1 (0.74)	-1.8 (<0.001)	-0.2 (0.4)	+0.6 (0.01)
Prolapse (n)	92152	92536	95888	97799	84956	92299	100561
Surgical care (%)	12.2	12.1	12.2	11.9	9.2	9.7	11.5
Δ %[a] (*P*-value)		-0.1 (0.51)	+0.1 (0.51)	-0.3 (0.04)	-2.7 (<0.001)	+0.5 (<0.001)	+1.8 (<0.001)
First trimester pregnancy complications (n)	39395	36248	37129	35809	34908	35323	33749
Surgical care (%)	30.0	26.7	24.5	24.0	21.8	20.4	20.2
Δ %[a] (*P*-value)		-3.3 (<0.001)	-2.2 (<0.001)	-0.5 (0.12)	-2.2 (<0.001)	-1.4 (<0.001)	-0.2 (0.52)
Infertility treatment (n)	60884	60562	60806	60583	57383	59801	54362
Surgical care (%)	2.5	2.4	2.0	1.9	1.5	1.5	1.5
Δ %[a] (*P*-value)		-0.1 (0.26)	-0.4 (<0.001)	-0.1 (0.22)	-0.4 (<0.001)	-0 (1.0)	-0 (1.0)
**TOTAL (N)**	320157	319503	331632	334575	309631	331109	335006
Surgical care (%)	13.3	12.7	12.4	11.9	10.1	10.1	10.9
Δ %[a] (*P*-value)		-0.6 (<0.001)	-0.3 (<0.001)	-0.5 (<0.001)	-1.8 (<0.001)	-0 (1.0)	+0.8 (<0.001)

= Covid-19 period

[a] = percentual difference from previous year

For peer review only - http://bmjopen.bmj.com/site/about/guidelines.xhtml

Overall, patient records increased from 320,157 in 2016 to 335,006 in 2022, representing a 4.6% rise. The highest number of records was in 2022 (335,006), while the lowest was in 2020 (309,631). The prolapse pathway had the largest volume, while endometriosis had the smallest. In 2020, the proportion of surgical care records declined across all pathways compared to 2019. The greatest decrease was in the prolapse pathway (-2.7%, $P<0.001$), while the smallest was in the infertility treatment pathway (-0.4%, $P<0.001$).

Figure 1 illustrates total patient records for surgical and non-surgical care from 2016 to 2022, with a predicted trendline from 2020 to 2022.

The predicted trendlines reveal a decrease in actual patient numbers compared to predicted patient numbers for both surgical- and non-surgical procedures in the year 2020 compared to pre-COVID trends. The drop in 2020 amounts to a reduction of 6% ($P=0.02$) for non-surgical care group patient numbers and a reduction of 18.3% for surgical care ($P<0.001$ and compared to non-surgical care $P=0.001$). In the non-surgical care group patient numbers were restored to predicted levels by July 2020. In the surgical care group numbers remained below predicted trends throughout 2020 and 2021 and returned to the predicted trendline in 2022. A seasonal fluctuation is evident in the surgical care group over the years.

Figure 2 present graphs of the six care pathways separately. The graphs show the number of patient records for both surgical- and nonsurgical care from 2016 to 2022, with a predicted trendline from 2020 to 2022.

A similar trend pattern was observed across the pathways for heavy menstrual bleeding, endometriosis, uterine fibroids, and prolapse. In these pathways, both surgical and non-

For peer review only

surgical procedures showed percentage decrease during the COVID-19 period in 2020. Over time, surgical care did not return to pre-COVID trends, whereas non-surgical care did. In the pathway heavy menstrual bleeding, the surgical procedures declined with 17.5% (*P*<0.001 and compared to non-surgical care *P*=0.01) while non-surgical procedures decreased by 5.5%, though not statistically significant (*P*=0.09). In the uterine fibroids pathway surgical care was reduced by 12.6% (*P*<0.001 and compared to non-surgical care *P*=0.2) and nonsurgical care was reduced by 7.5% (*P*=0.01). Both types of care in the endometriosis pathway dropped in percentages: surgical care by 16.9% (*P*=0.08 and compared to non-surgical care *P*=0.1) and non-surgical care by 5.4% (*P*=0.03). The largest decrease is seen in the pathway prolapse, with a decline of 10.4% (*P*=0.001) in non-surgical procedures and 17.5% (*P*<0.001 and compared to non-surgical care *P*=0.6) in surgical procedures.

The pathways for first trimester pregnancy complications and infertility treatment exhibited a pre-COVID pattern of declining surgical procedures and stable non-surgical procedures. During the initial COVID-19 period, both types of care declined, but they returned to pre-COVID trends by around June/July 2020. The first trimester pregnancy complications pathway shows a non-significant difference of 0.6% (*P*=0.8) of non-surgical procedures over 2020 compared to predicted trends, while the surgical procedures declined with 17.7% (*P*<0.001 and compared to non-surgical care *P*=0.2). Infertility treatment shows a similar pattern with a non-significant 2.2% decrease in non-surgical procedures (*P*=0.6) and a statistically significant 19.3% reduction in surgical procedures (*P*<0.001 and compared to non-surgical care *P*=0.01).

The percentages and P-values reported above are derived from regression models that adjust for time trends, intervention type (surgical vs. non-surgical), and the specific impact of

For peer review only - http://bmjopen.bmj.com/site/about/guidelines.xhtml

the year 2020 (COVID-19). Full model coefficients and interaction terms are presented in Table 3.

Table 3. Regression model output for yearly trends and COVID-related changes in surgical and non-surgical care across the six gynecological care pathways.

Model:	Total[a]	Heavy menstrual bleeding[a]	Uterine fibroids[a]	Endometriosis[b]	Prolapse[b]	Infertility treatment[a]	First trimester pregnancy complications[b]
Variables:							
	10.053**	8.772**	7.353**		8.819**	8.522**	
Intercept	(0.016)	(0.020)	(0.018)	7.093**		(0.026)	7.729**
	0.013**	0.012*	0.011			-0.013	
Year	(0.005)	(0.006)	(0.005)	0.106**	0.012**	(0.007)	0.001
	-1.878**	-1.767**	-1.539**			-3.677**	
OR_prestation	(0.023)	(0.028)	(0.026)	-1.068**	-2.789**	(0.037)	-1.707**
	-0.046**	-0.042**	-0.015*			-0.096**	
Int_Year_OR	(0.007)	(0.008)	(0.007)	-0.139**	-0.045*	(0.011)	-0.031
	-0.060*	-0.055	-0.075*			-0.022	
Dummy_2020	(0.026)	(0.032)	(0.029)	-0.054	-0.104**	(0.043)	-0.006
	-0.123**	-0.119**	-0.051			-0.171**	
Int_OR_2020	(0.037)	(0.045)	(0.041)	-0.115	-0.071	(0.060)	-0.170

Notes: OR = operating room (surgical care).
Intercept: baseline level in 2016;
Year: time trend;
OR_prestation: effect of surgical care (OR) vs. non-surgical care (1 = surgical);
Int_Year_OR: interaction Year × OR_prestation (surgical care);
Dummy_2020: effect of year 2020 (COVID-19);
Int_OR_2020: interaction OR_prestation × 2020.

[a] = linear regression model normally distributed
[b] = linear regression model with non-parametric bootstraps R=10,000
Standard errors in parentheses
Significance codes: **= <0.01, *= <0.05

For peer review only

DISCUSSION

In this quantitative cohort study, we evaluated the potential substitution of surgical procedures with non-surgical procedures across six gynaecological care pathways during the initial phase of the COVID-19 pandemic in the Netherlands. Our analysis revealed an 18.3% reduction in surgical and 6% in non-surgical care across all pathways in 2020. While non-surgical procedures resumed pre-pandemic trends by mid-2020, surgical procedures remained below baseline. This implies that reductions in healthcare in 2020 were not compensated during the duration of the study. This trend was most pronounced in the prolapse pathway and was also observed in the pathways for menstrual disorders, uterine fibroids, and endometriosis.

In contrast, the care pathways for urgent first trimester pregnancy complications and infertility treatment showed pre-pandemic trends of declining surgical procedures and stable non-surgical procedures. For first trimester pregnancy complications, reductions were minimal, likely due to the urgent character of care, mirroring patterns for acute care during the pandemic.[11, 33, 34] Infertility care decreased early in the pandemic, with both procedural types returning to pre-pandemic patterns by mid-2020.

Our findings align with literature documenting reductions in elective surgical care. Previous studies attributed these to the redeployment of healthcare resources towards COVID-19 care and the imposition of restrictions on elective procedures.[35] For example, a study in Japan reported an 8.5% decline in benign gynaecological surgeries in 2020 compared to 2019 [18], while New South Wales experienced a 37% reduction in general elective surgeries.[33]

Contrary to our initial hypothesis, we found no increase in substitution by non-surgical alternatives. Instead, both surgical and non-surgical procedures declined across multiple

For peer review only - http://bmjopen.bmj.com/site/about/guidelines.xhtml

care pathways. This aligns with earlier reports of reduced non-COVID medical and surgical hospitalizations by 22% and 33%, respectively [36], suggesting a more generalized reduction in healthcare capacity during the pandemic. This overall decrease may have resulted from a combination of reduced patient demand and reduced healthcare capacity. Data from the Dutch Healthcare Authority and ZorgDomein showed that the number of referrals during the first covid wave was 12% lower than in 2019, with a marked decline in gynaecology referrals between March and May 2020, before returning to normal by June.[37] To our surprise no increase of referrals was observed to recover delayed care. This decline in referrals contributed to the observed reduction in both surgical and non-surgical procedures within the gynaecological care pathways [19], but does not explain the sustained effect of continuously lower surgical procedure rates.

Patient-related factors likely influenced the reduction in referrals during the pandemic. Older patients, at higher risk for severe COVID-19 outcomes [38], may have been more hesitant to seek care, which could explain the pronounced decline in the prolapse pathway, predominantly affecting older individuals. Symptom severity and urgency also played a role; public health campaigns discouraged in-person visits [39-41], likely deterring patients from seeking care for non-urgent conditions such as menstrual disorders, fibroids, endometriosis, and prolapse. Although stay-at-home measures may have increased awareness of symptoms, they may also have allowed patients to tolerate these symptoms more easily in a home-based setting, reducing help-seeking behaviour.

The fact that care volumes did not return to pre-pandemic levels in 2021 or 2022 suggests that much of the missed care was not simply ‘’caught up’’. The persistent declines across all six care pathways studied may reflect structural changes in care delivery. Patients may have

For peer review only - http://bmjopen.bmj.com/site/about/guidelines.xhtml

deferred or abandoned treatment for various reasons, such as symptom resolution, fluctuating symptoms, or changing perceptions of the risks and benefits of surgery in the aftermath of the pandemic. In the case of prolapse, mortality in older patients may also have played a role. For abnormal bleeding, the natural onset of menopause may have led to symptom reduction. In addition, organizational constraints such as ongoing capacity limitations, staff shortages, and prioritization of other care domains may have contributed to sustained lower care volumes. These findings suggest that the pandemic not only caused temporary disruption in 2020 but may have accelerated or reinforced shifts in gynaecological care patterns that require long-term follow-up. Understanding whether these changes reflect appropriate care reduction, unmet need, or both is essential for future planning in benign gynaecology.

Professional preferences and perceptions may have contributed to treatment deferral. Gynaecologists may perceive non-surgical options as less effective than surgical procedures. For instance, uterine artery embolization may be viewed as less reliable than hysterectomy, prompting delays until surgery was possible.[42] Previous research indicates that not all gynaecologists discuss uterine embolization as a treatment for fibroids.[43] When surgical procedures are considered the gold standard, particularly for conditions like prolapse, fibroids, or advanced endometriosis, gynaecologists may recommend delaying treatment rather than pursuing less effective non-surgical alternatives.

This inclination to defer surgery may be inappropriate in moderate to severe cases like prolapse or endometriosis, where symptom progression is likely. According to the Dutch Association of Medical Specialists (2023), patient preferences also influence treatment decisions.[44] Some patients prefer to wait for definitive surgery after failed conservative

For peer review only - http://bmjopen.bmj.com/site/about/guidelines.xhtml

treatments or view non-surgical options as temporary, reinforcing a shared decision to prioritize surgery even in times of resource scarcity.

At the system level, pandemic-related staff redeployment, including anaesthesiologists to COVID-19 units, reduced operating room capacity for elective procedures.[5] Gynaecological procedures like those for menstrual disorders, fibroids, and prolapse were deferred, while urgent surgeries, such as those for first-trimester pregnancy complications, continued due to their non-elective nature. COVID-19-related staff absences also delayed elective surgeries and outpatient intakes.[45] Combined with patient reluctance and perceptions of non-surgical alternatives, these factors likely drove the significant procedural volume reductions observed.

Strengths and limitations

A major strength is our large dataset spanning all benign gynaecological elective care in Dutch hospitals and clinics over six years. This allows a detailed longitudinal insight into gynaecological care trends before, during and after the COVID-19 pandemic. Moreover, the Dutch Hospital Data (DHD) provider follows standardized data collection protocols that enhance the comparability of our findings. In addition, examining multiple gynaecological care pathways offers a detailed understanding of how different clinical contexts were affected by the pandemic.

Nevertheless, there are important limitations. The observational nature of the study limits our ability to infer causality between the pandemic and the changes in care utilization. Additionally, we did not assess primary health care use, which might have substituted a share of the secondary care, and patient outcomes, which restricts our ability to evaluate the effectiveness of the reduced or substituted non-surgical procedures. This latter

For peer review only - http://bmjopen.bmj.com/site/about/guidelines.xhtml

For peer review only

limitation is particularly relevant when considering the long-term consequences of deferred surgical interventions.

Implications for practice

Our findings have significant implications for clinical practice and healthcare policy. The observed reduction in both surgical and non-surgical procedures during the initial phase of the COVID-19 pandemic, with no evidence of subsequent compensation, suggests a fundamental shift in healthcare delivery: care was not deferred or substituted, but it appears to have vanished. This phenomenon of forgone care necessitates a deeper understanding of the underlying causes and the potential long-term consequences for patients and healthcare systems.

First, it is critical to investigate what happened to the patients who did not receive care during this period. Did they seek alternative forms of care, such as treatment in primary care settings or through conservative management approaches? Or did they forego care altogether, potentially leading to unresolved health issues or adverse outcomes? Understanding these dynamics is essential to ascertain whether this reduction in care resulted in unmet medical needs or whether it represents an unintentional shift toward more appropriate care delivery. Such insights could provide valuable context for assessing whether these changes align with principles of necessity and proportionality in healthcare provision.

Moreover, the long-term effects of this vanished care must be studied in detail. Questions remain as to whether the reduced volume of both surgical and non-surgical procedures reflects changes on the demand side during crisis, e.g. in disease incidence, patient preferences, or systemic barriers to access. Alternatively, the crisis might have affected the

For peer review only - http://bmjopen.bmj.com/site/about/guidelines.xhtml

use of care through supply side changes. Was the decline driven by restricted health care capacity: resource limitations, workforce shortages, or a broader recalibration of clinical priorities during the pandemic? Understanding these drivers will inform strategies to balance immediate care demands with the need to ensure equitable access in future crises.

A better understanding might also help to define the appropriateness of care in non-crisis settings. The COVID-19 pandemic created a unique opportunity to critically evaluate which procedures are essential and which may be of limited benefit. However, the observed reduction in care must not be universally interpreted as progress towards more appropriate care.

For peer review only

CONCLUSION

The COVID-19 pandemic significantly disrupted both surgical and non-surgical procedures within benign gynaecology pathways. Reduced care uptake during COVID waves was not recovered, but forgone. The reduction in surgical procedures did not correspond with increased use of non-surgical alternatives. This raises important societal questions about consequences of unused care and especially whether the reduction in surgical procedures is desirable or has left patients with a disproportionately reduced quality of life.

Future research should prioritize evaluating the impact of this disruption at both patient and societal level. Qualitative research is needed to understand the individual patient experiences and outcomes, while quantitative studies should assess broader implications for care pathways and healthcare costs. Our findings offer valuable insights that can guide healthcare policy and practice, emphasizing the importance of developing resilient systems capable of delivering high-quality care in the face of future disruptions and relative scarcity of medical capacity.

For peer review only

Data sharing statement

The data used for this study were obtained from Statistics Netherlands (Centraal Bureau voor de Statistiek, CBS) under a data-sharing agreement. Due to privacy regulations and the confidentiality of individual-level data, the dataset cannot be shared publicly. Access to CBS data is restricted and requires approval through their secure microdata services, available to registered institutions under specific conditions.

Authorship statement

All authors made substantial contributions to the conception, design, and acquisition of data for the work, as well as the analysis and interpretation of the data. E.L.M. Velthuijs and I. Ismail had a particularly significant role in the analysis of the data. E.L.M. Velthuijs drafted the manuscript, with critical revisions from I. Ismail, A.H.E. Koolman, R.A. de Leeuw, and W.J.K. Hehenkamp. All authors reviewed the manuscript critically for important intellectual content. Each author gave final approval of the version to be published and agrees to be accountable for all aspects of the work, ensuring that questions related to the accuracy or integrity of any part of the work are appropriately investigated and resolved. Eva Velthuijs is the guarantor for this paper.

Competing interest statement

None of the authors have a potential conflict of interest.

Funding

This study was funded by ZonMw, the Dutch Organization for Health Research and Development, under project number 05160482240003.

For peer review only

For peer review only - http://bmjopen.bmj.com/site/about/guidelines.xhtml

Research Ethics Approval

This study involves human participants but was not approved by an Ethics Committee(s) or Institutional Board(s). We used CBS microdata for our research. As part of the data application process, CBS assesses whether our study complies with legal and ethical guidelines. This review ensures that our research meets ethical standards before access to the data is granted. Since our study is retrospective and based on fully anonymized data, it does not fall under the scope of the Medical Research Involving Human Subjects Act (WMO), and approval from an external Medical Ethics Review Committee (METC) is not required.

Participant consent statement

Not required. The study used retrospective, fully anonymised data from CBS microdata. In accordance with Dutch law and institutional policies, informed consent is not necessary for research using de-identified routinely collected health data.

For peer review only

For peer review only - http://bmjopen.bmj.com/site/about/guidelines.xhtml

REFERENCES

1. OECD. Ready for the Next Crisis? Investing in Health System Resilience. 2023.

2. Cynthia Cox JO, Emma Wager, Krutika Amin. Health Care Costs and Affordability. KFF; 2024.

3. Adan I, Bekkers J, Dellaert N, et al. Improving operational effectiveness of tactical master plans for emergency and elective patients under stochastic demand and capacitated resources. European Journal of Operational Research. 2011;213(1):290-308.

4. Cowie J, Nicoll A, Dimova ED, et al. The barriers and facilitators influencing the sustainability of hospital-based interventions: a systematic review. BMC Health Services Research. 2020;20(1):588.

5. CMS. Adult Elective Surgery and Procedures Recommendations:

Limit all non-essential planned surgeries and procedures, including dental, until further notice. 2020.

6. Francis N, Dort J, Cho E, et al. SAGES and EAES recommendations for minimally invasive surgery during COVID-19 pandemic. Surgical Endoscopy. 2020;34(6):2327-31.

7. Pryor A. SAGES and EAES Recommendations Regarding Surgical Response to COVID-19 Crisis 2020 [Available from: https://www.sages.org/recommendations-surgical-response-covid-19/.

8. COVIDSurgCollaborative. Global guidance for surgical care during the COVID-19 pandemic. British Journal of Surgery. 2020;107(9):1097-103.

For peer review only - http://bmjopen.bmj.com/site/about/guidelines.xhtml

9. Moynihan R, Sanders S, Michaleff ZA, et al. Impact of COVID-19 pandemic on utilisation of healthcare services: a systematic review. BMJ Open. 2021;11(3):e045343.

10. de Graaff MR, Hogenbirk RNM, Janssen YF, et al. Impact of the COVID-19 pandemic on surgical care in the Netherlands. Br J Surg. 2022;109(12):1282-92.

11. FederatieMedischSpecialisten. Leidraad om veilige non-COVID-zorg in ziekenhuizen ten tijde van de verschillende SARS-CoV-2 epidemiologische fases mogelijk te maken. 2020.

12. Winkelmann J, Webb E, Williams GA, et al. European countries' responses in ensuring sufficient physical infrastructure and workforce capacity during the first COVID-19 wave. Health Policy. 2022;126(5):362-72.

13. Moynihan R, Johansson M, Maybee A, et al. Covid-19: an opportunity to reduce unnecessary healthcare. BMJ. 2020;370:m2752.

14. Oosterhoff M, Kouwenberg LHJA, Rotteveel AH, et al. Estimating the health impact of delayed elective care during the COVID -19 pandemic in the Netherlands. Social Science & Medicine. 2023;320:115658.

15. van Giessen A, de Wit A, van den Brink C, et al. Impact van de eerste COVID-19 golf op de reguliere zorg en gezondheid : Inventarisatie van de omvang van het probleem en eerste schatting van gezondheidseffecten. Impact of the first wave of COVID-19 on regular healthcare and health : Assessment of the extent of the problem and first estimation of the health effects: Rijksinstituut voor Volksgezondheid en Milieu RIVM; 2020.

16. Diaz A, Sarac BA, Schoenbrunner AR, et al. Elective surgery in the time of COVID-19. The American Journal of Surgery. 2020;219(6):900-2.

For peer review only - http://bmjopen.bmj.com/site/about/guidelines.xhtml

17. Spinelli A, Pellino G. COVID-19 pandemic: perspectives on an unfolding crisis. British Journal of Surgery. 2020;107(7):785-7.

18. Komatsu H, Nakagawa S, Yamagami W, et al. Impact of the COVID-19 pandemic on surgery for benign diseases in gynecology: A nationwide survey by the Japan Society of Obstetrics and Gynecology. Journal of Obstetrics and Gynaecology Research. 2023;49(1):314-20.

19. Nederlandse Zorgautoriteit. Analyse van de gevolgen van de coronacrisis voor de reguliere ziekenhuiszorg - 26 mei 2020. 2020.

20. Rahman R, Gupta S, Manyonda I. Hysterectomy for benign gynaecological disease. Obstetrics, Gynaecology & Reproductive Medicine. 2017;27(4):125-31.

21. Vink Maarten D.H.P., France R.M., van Wezep Tim C., et al. Practice variation in the stepped care approach to idiopathic heavy menstrual bleeding: A population-based study. European Journal of Obstetrics & Gynecology and Reproductive Biology; 2023. p. 6-12.

22. FederatieMedischSpecialisten. Handreiking Implementatie Passende zorg Bouwstenen voor de praktijk. demedischspecialist.nl2023.

23. Cohen ES, Kouwenberg LHJA, Moody KS, et al. Environmental sustainability in obstetrics and gynaecology: A systematic review. BJOG: An International Journal of Obstetrics & Gynaecology. 2024;131(5):555-67.

24. WorldHealthOrganizationSecretariat. Global strategy on human resources for health: Workforce 2030. Geneva: WHO; 2016.

25. Burki T. Global shortage of personal protective equipment. The Lancet Infectious Diseases. 2020;20(7):785-6.

For peer review only

26. Abdool Z, Thakar R, Sultan AH, et al. Prospective evaluation of outcome of vaginal pessaries versus surgery in women with symptomatic pelvic organ prolapse. International Urogynecology Journal. 2011;22(3):273-8.

27. Brennan A, Hickey M. Abnormal uterine bleeding: managing endometrial dysfunction and leiomyomas. Medical Journal of Australia. 2018;208(2):90-5.

28. Kalaitzopoulos DR, Samartzis N, Kolovos GN, et al. Treatment of endometriosis: a review with comparison of 8 guidelines. BMC Women's Health. 2021;21(1):397.

29. Matteson KA, Abed H, Wheeler TL, et al. A systematic review comparing hysterectomy with less-invasive treatments for abnormal uterine bleeding. J Minim Invasive Gynecol. 2012;19(1):13-28.

30. Rezende GP, Venturini MC, Kawagoe LN, et al. Surgery vs. hormone-based treatment for pain control in deep infiltrating endometriosis: a retrospective cohort study. Curr Med Res Opin. 2022;38(4):641-7.

31. van der Kooij SM, Bipat S, Hehenkamp WJK, et al. Uterine artery embolization versus surgery in the treatment of symptomatic fibroids: a systematic review and metaanalysis. American Journal of Obstetrics and Gynecology. 2011;205(4):317.e1-.e18.

32. van der Vaart LR, Vollebregt A, Milani AL, et al. Pessary or surgery for a symptomatic pelvic organ prolapse: the PEOPLE study, a multicentre prospective cohort study. Bjog. 2022;129(5):820-9.

33. McBride KE, Steffens D, Solomon MJ. From the sidelines: The indirect repercussions of COVID-19 on the delivery of hospital surgical services. ANZ J Surg. 2021;91(7-8):1345-51.

For peer review only - http://bmjopen.bmj.com/site/about/guidelines.xhtml

34. Nederlandse Zorgautoriteit. Analyse van de gevolgen van de coronacrisis voor de reguliere zorg 2020.

35. Mayol J, Fernández Pérez C. Elective surgery after the pandemic: waves beyond the horizon. British Journal of Surgery. 2020;107(9):1091-3.

36. Gasch-Illescas A, Calle-Serrano M, Vallejo-Vaz AJ, et al. Impact of the first wave of the COVID-19 pandemic on non-COVID inpatient care in southern Spain. Sci Rep. 2023;13(1):1634.

37. Zorgdomein. Uitgestelde zorg loopt in 2020 op tot ruim 1,7 miljoen verwijzingen2020. Available from: https://zorgdomein.com/nieuws/uitgestelde-zorg-loopt-in-2020-op-tot-ruim-17-miljoen-verwijzingen/.

38. CDC. Underlying Medical Conditions Associated with Higher Risk for Severe COVID-19: Information for Healthcare Professionals 2021 [updated 24-4-2024. Available from: https://www.cdc.gov/coronavirus/2019-ncov/hcp/clinical-care/underlyingconditions.html.

39. Dupraz J, Le Pogam M-A, Peytremann-Bridevaux I. Early impact of the COVID-19 pandemic on in-person outpatient care utilisation: a rapid review. BMJ Open. 2022;12(3):e056086.

40. Green MA, McKee M, Katikireddi SV. Remote general practitioner consultations during COVID-19. The Lancet Digital Health. 2022;4(1):e7.

41. Jaffe TA, Hayden E, Uscher-Pines L, et al. Telehealth use in emergency care during coronavirus disease 2019: a systematic review. Journal of the American College of Emergency Physicians Open. 2021;2(3):e12443.

For peer review only - http://bmjopen.bmj.com/site/about/guidelines.xhtml

42. Gupta JK, Sinha A, Lumsden MA, et al. Uterine artery embolization for symptomatic uterine fibroids. Cochrane Database Syst Rev. 2012(5):Cd005073.

43. de Bruijn AM, Huisman J, Hehenkamp WJK, et al. Implementation of uterine artery embolization for symptomatic fibroids in the Netherlands: an inventory and preference study. CVIR Endovasc. 2019;2(1):18.

44. FederatieMedischSpecialisten. Richtlijn Hevig menstrueel bloedverlies (HMB). Richtlijnendatabase; 2023.

45. Nederlandse Zorgautoriteit. Monitor Toegankelijkheid van Zorg; gevolgen van Covid-19 - 26 augustus 2021. 2021.

For peer review only

Figures

Title:

Figure 1. Total patient count surgical- and nonsurgical care timeline 2016-2022 for the 6 care pathways combined [a]

Legend:

[a] Trendlines are presented using three-months moving averages

[b] Regression models were used to estimate deviations from expected trends in the year 2020; p-values for this period's effect are reported within the graph in corresponding colour, full model can be found in *Table 3*.

Title:

Figure 2. Six care pathways surgical- and nonsurgical care timeline 2016-2022 [a]

Legend:

[a] Trendlines are presented using three-months moving averages

[b] Regression models were used to estimate deviations from expected trends in the year 2020; p-values for this period's effect are reported within the graphs in corresponding colour, full models can be found in *Table 3*.

For peer review only - http://bmjopen.bmj.com/site/about/guidelines.xhtml

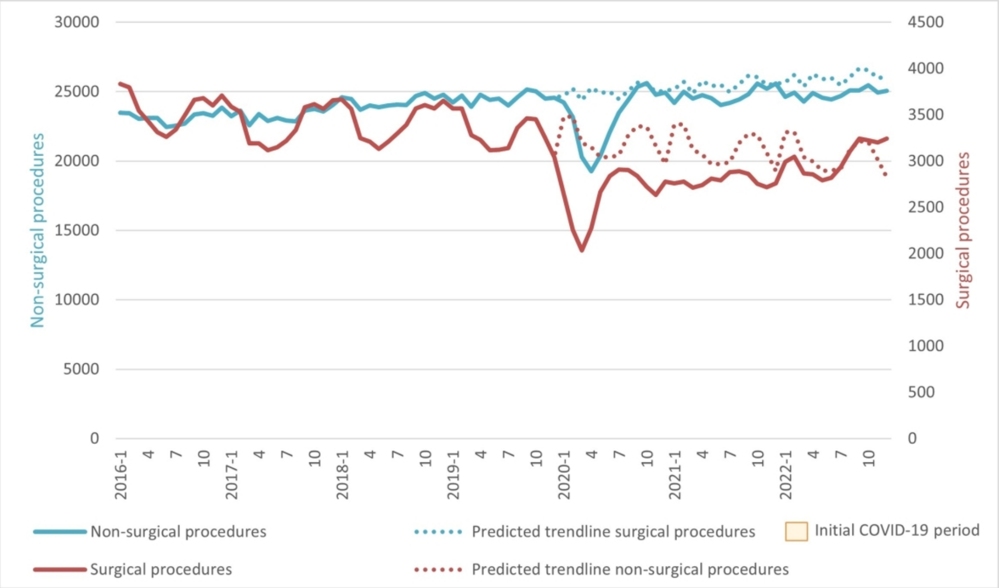

Caption:
Figure 1. Total patient count surgical- and nonsurgical care timeline 2016-2022 for the 6 care pathways combined a
Legend:
a Trendlines are presented using three-months moving averages
b Regression models were used to estimate deviations from expected trends in the year 2020; p-values for this period's effect are reported within the graph in corresponding colour, full model can be found in Table 3.

178x105mm (300 x 300 DPI)

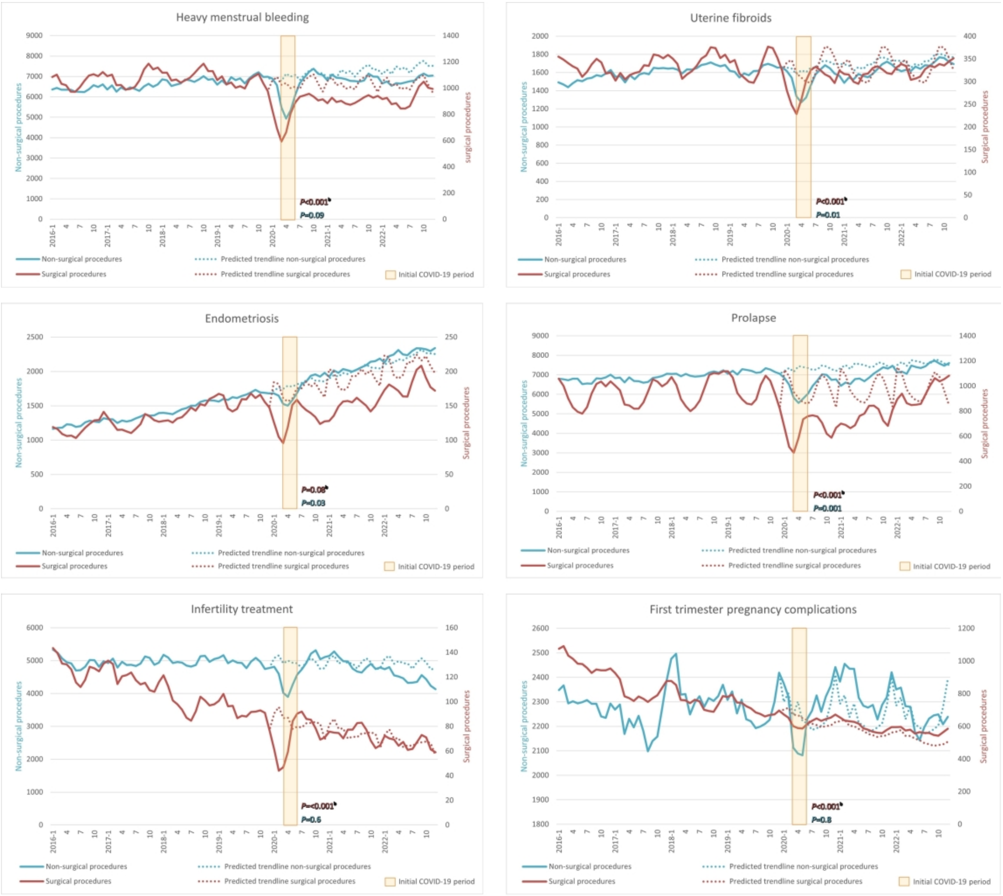

Caption:
Figure 2. Six care pathways surgical- and nonsurgical care timeline 2016-2022 a
Legend:
a Trendlines are presented using three-months moving averages
b Regression models were used to estimate deviations from expected trends in the year 2020; p-values for this period's effect are reported within the graphs in corresponding colour, full models can be found in Table 3.

210x198mm (300 x 300 DPI)
